# Cardiomyopathy Following Olanzapine Use in an Octogenarian: A Case Report and Literature Review

**DOI:** 10.7759/cureus.114285

**Published:** 2026-08-10

**Authors:** Sahar S Abdelmoneim, Priscila Sole, Manuel De la Cruz Seoane, Ismael Palacio, Leosbel Hurtado, Pedro Valdes, Brian Valle

**Affiliations:** 1 Cardiovascular Medicine, Larkin Hospital Palm Spring Campus, Hialeah, USA; 2 General Internal Medicine/Cardiovascular Medicine, Assiut University Hospital, Assiut, EGY; 3 Critical Care Medicine, The University of Edinburgh, Edinburgh, GBR; 4 Medicine, Centro Universitário Lusíada, Santos, BRA; 5 Internal Medicine, Larkin Community Hospital, Miami, USA

**Keywords:** adverse drug reaction, antipsychotic cardiotoxicity, cardiomyopathy, drug-induced cardiomyopathy, heart failure, left ventricular dysfunction, nonischemic cardiomyopathy, olanzapine, older adults, polypharmacy

## Abstract

Olanzapine is a widely prescribed second-generation antipsychotic. We report the case of an 81-year-old woman with hypertension, type 2 diabetes mellitus, osteoporosis, and long-term olanzapine use who presented with progressive dyspnea, orthopnea, paroxysmal nocturnal dyspnea, lower extremity edema, and palpitations. Transthoracic echocardiography demonstrated four-chamber dilation, global left ventricular hypokinesis, and a reduced left ventricular ejection fraction (LVEF) of 30-35%. Coronary angiography revealed normal coronary arteries. After initiation of guideline-directed medical therapy for heart failure and discontinuation of olanzapine because of suspected drug-associated cardiomyopathy, the patient clinically stabilized. At outpatient follow-up, transthoracic echocardiography demonstrated partial recovery of left ventricular systolic function, with the LVEF improving to 40.77%. This case emphasizes the necessity of considering medication-associated cardiomyopathy in the differential diagnosis of new-onset nonischemic heart failure, particularly in patients receiving long-term antipsychotic therapy. Early recognition of cardiotoxicity and withdrawal of the offending agent may improve clinical outcomes.

## Introduction

As the global older adult population continues to grow, multimorbidity and polypharmacy have become increasingly prevalent, contributing to adverse cardiovascular outcomes and greater complexity in clinical management. Polypharmacy is common among older adults and has been associated with adverse drug events, medication nonadherence, poorer health outcomes, increased hospitalizations, and higher healthcare costs [1].

Among the medications frequently associated with unexpected side effects are antipsychotics. While second-generation antipsychotics are widely used to manage late-life psychiatric disorders, they are known to cause cardiovascular adverse effects, ranging from orthostatic hypotension and QT interval prolongation to myocarditis and sudden cardiac death. In addition, they may contribute to metabolic disturbances such as weight gain, dyslipidemia, and diabetes mellitus, which further increase cardiovascular risk. Beyond these established metabolic and electrophysiological risks, emerging evidence suggests a potential association between olanzapine use and nonischemic cardiomyopathy. Proposed mechanisms of olanzapine-associated cardiac injury include direct myocardial toxicity, inflammatory and oxidative pathways, mitochondrial dysfunction, and indirect metabolic effects, although the underlying pathophysiology remains incompletely understood [1-4].

Management of polypharmacy requires a multidisciplinary approach that includes regular medication review, optimization of nonpharmacologic interventions when appropriate, shared decision-making with patients and caregivers, and close interprofessional communication to improve medication safety and clinical outcomes. Maintaining a broad differential diagnosis is particularly important in older adults presenting with new-onset heart failure, especially in the setting of chronic antipsychotic exposure [1-3].

Here, we report a case of heart failure potentially associated with long-term olanzapine use in an octogenarian, followed by a review of the literature. Given the limited number of published cases of olanzapine-associated cardiotoxicity, this case adds to the scarce clinical evidence, particularly regarding prolonged exposure in older adults, in whom competing etiologies may complicate causal assessment.

## Case presentation

We present an 81-year-old woman with a history of bipolar disorder, hypertension, type 2 diabetes mellitus, and osteoporosis who presented to the emergency department with a 15-day history of progressive shortness of breath. She reported orthopnea, paroxysmal nocturnal dyspnea, dyspnea on exertion, bilateral lower extremity edema, and fatigue. Her home medications included alendronate, amlodipine, aspirin, glimepiride, losartan, metoprolol, and olanzapine 5 mg daily, which she had reportedly taken for more than 10 years for bipolar disorder.

She denied any prior similar episodes, recent viral illness, myocardial infarction, stroke, or deep venous thrombosis. Two days prior to presentation, she had undergone an outpatient electrocardiogram that was reportedly normal. She lived independently, performed all activities of daily living, and had recently experienced the loss of her spouse.

On presentation, vital signs were stable. Cardiovascular examination was notable for an S3 gallop and 4+ bilateral lower-extremity edema. Initial laboratory studies (Table 1) demonstrated a markedly elevated B-type natriuretic peptide (BNP) level, mild anemia, and hyperglycemia. Serial troponin I measurements remained within the reference range, and renal, hepatic, thyroid, and electrolyte parameters were unremarkable. Urine drug screen was negative.

**Table 1:** Admission laboratory findings BNP: B-type natriuretic peptide, eGFR: estimated glomerular filtration rate, AST: aspartate aminotransferase, ALT: alanine aminotransferase, TSH: thyroid-stimulating hormone

Laboratory parameter	Admission value	Reference range
White blood cell count (×10³/µL)	7.1	4.0-10.0
Hemoglobin (g/dL)	11.7	12.0-16.0
Hematocrit (%)	36.3	36-46
Platelet count (×10³/µL)	176	150-400
Sodium (mmol/L)	139	135-145
Potassium (mmol/L)	4.4	3.5-5.0
Chloride (mmol/L)	98	98-107
Bicarbonate/CO₂ (mmol/L)	34	22-29
Blood urea nitrogen (mg/dL)	21	8-20
Creatinine (mg/dL)	0.82	0.6-1.1
eGFR (mL/min/1.73 m²)	81	≥60
Glucose (mg/dL)	208	70-99
Calcium (mg/dL)	9.4	8.4-10.2
Albumin (g/dL)	4.3	3.5-5.0
Total protein (g/dL)	7.7	6.0-8.3
AST (U/L)	23	10-40
ALT (U/L)	26	10-40
Total bilirubin (mg/dL)	1.0	0.2-1.2
TSH (mIU/L)	1.24	0.4-5.0
Magnesium (mg/dL)	2.3	1.6-2.6
BNP (pg/mL)	10,680	<100
Troponin I (ng/mL)	0.03	≤0.04

**Figure 1:**
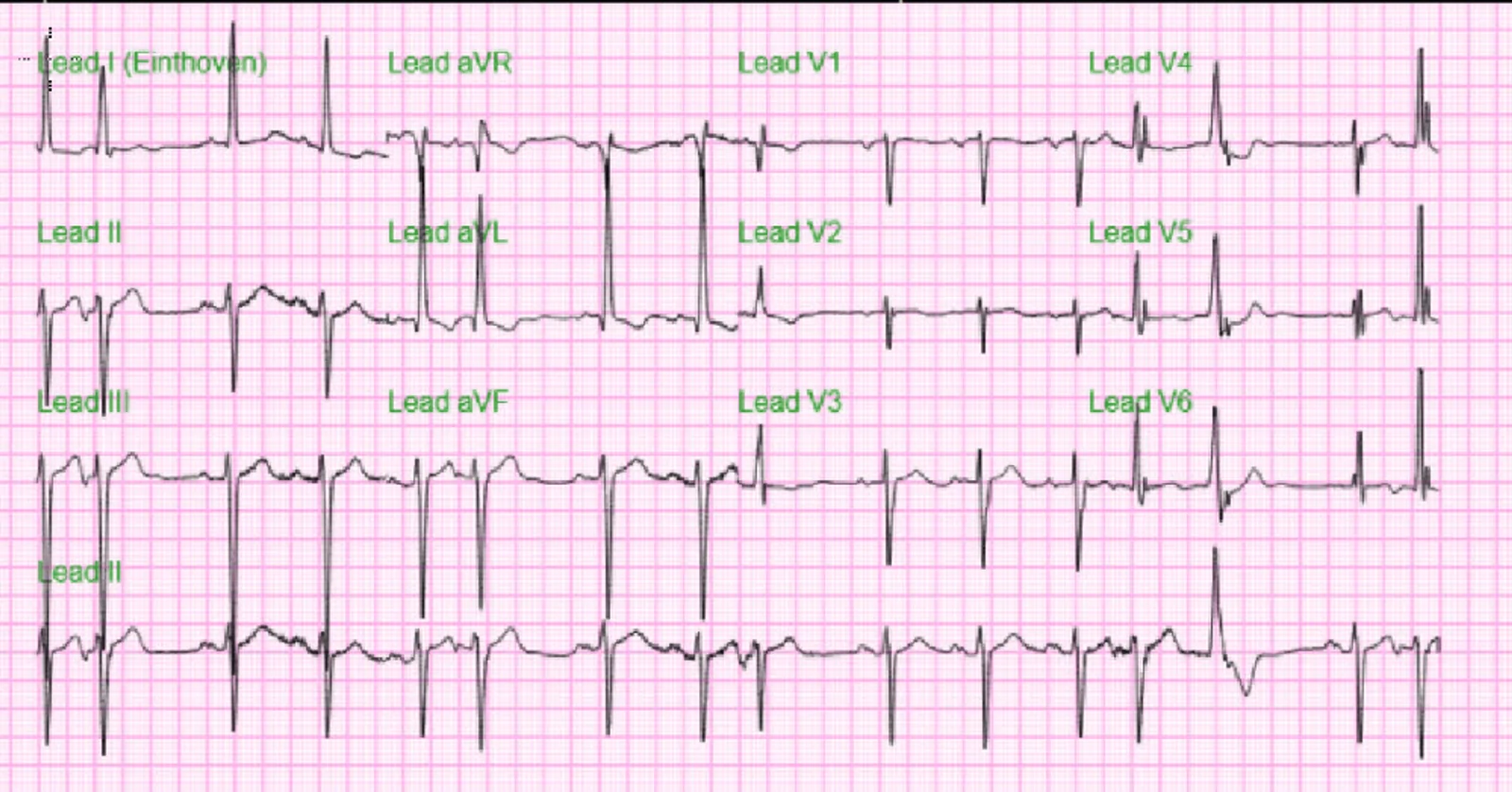
Admission electrocardiogram Electrocardiogram demonstrated sinus rhythm with frequent premature ventricular complexes and premature atrial contractions, without acute ST-segment elevation.

Electrocardiography demonstrated sinus rhythm with frequent premature ventricular complexes and premature atrial contractions (Figure 1).

The patient was admitted for further evaluation and management. Transthoracic echocardiography revealed global left ventricular hypokinesis, moderate diastolic dysfunction (pseudonormal filling pattern), four-chamber dilation, and a left ventricular ejection fraction (LVEF) of approximately 30-35%, as documented in the official echocardiography report. She was treated with intravenous furosemide and metoprolol for acute decompensated heart failure. Serial troponin measurements remained negative. BNP decreased to 4,690 pg/mL by hospital day 3. Renal function remained preserved throughout hospitalization, with serum creatinine ranging from 0.82 to 1.13 mg/dL.

**Figure 2:**
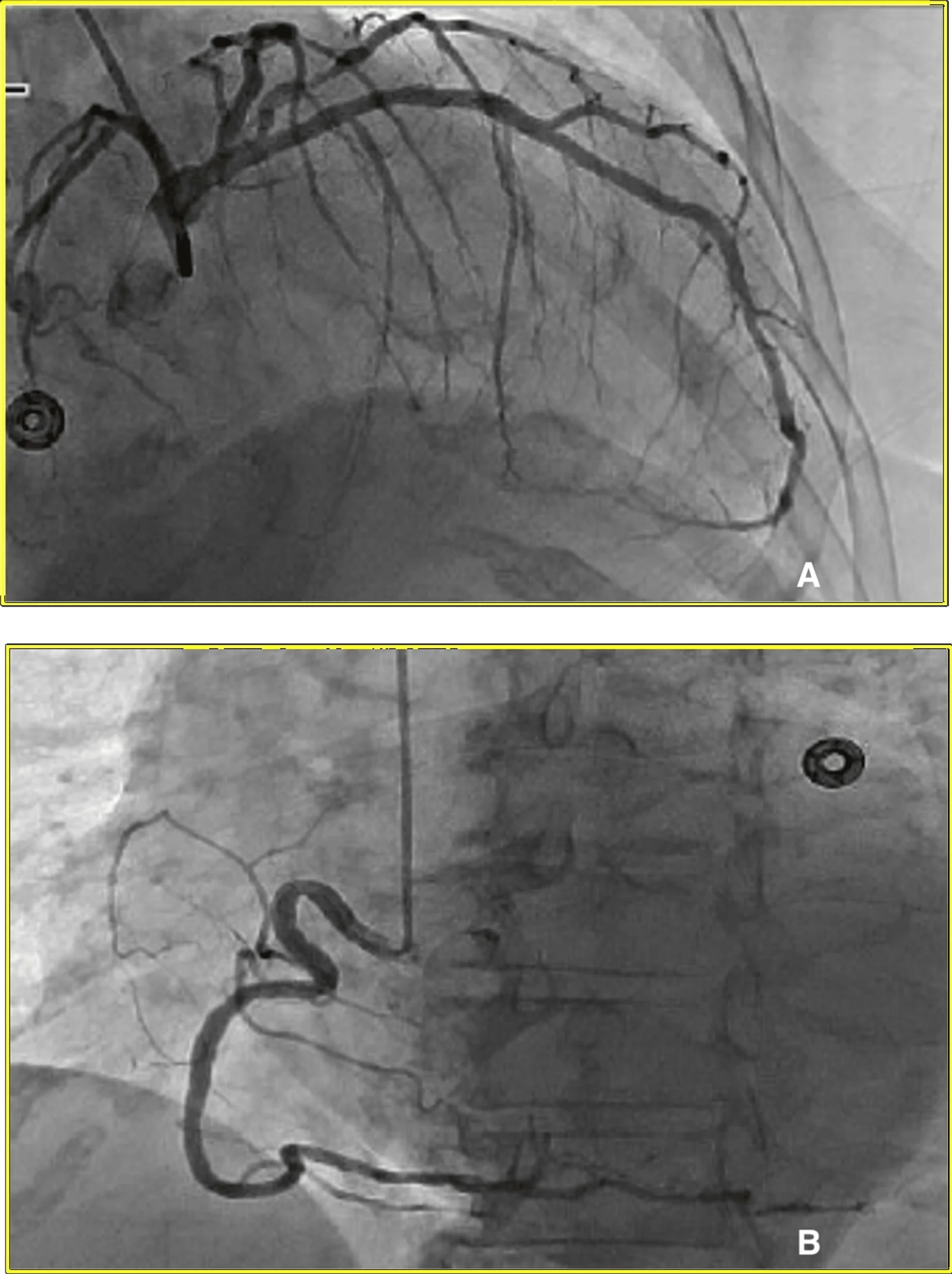
Coronary angiography (A) Left coronary angiography and (B) right coronary angiography demonstrating normal coronary arteries without significant obstructive coronary artery disease.

Cardiac catheterization with coronary angiography was performed on hospital day 5 to rule out an ischemic etiology. The angiogram demonstrated no significant obstructive coronary artery disease (Figure 2). Left ventriculography confirmed ventricular hypokinesis with an estimated LVEF of 30-35%.

Following coronary angiography, the patient was diagnosed with nonischemic dilated cardiomyopathy. The differential diagnosis included stress-induced (Takotsubo) cardiomyopathy and medication-associated cardiomyopathy. Given the absence of obstructive coronary artery disease, the lack of a clear alternative etiology, and the history of prolonged olanzapine exposure, olanzapine-associated cardiomyopathy was considered. Olanzapine was discontinued.

**Figure 3:**
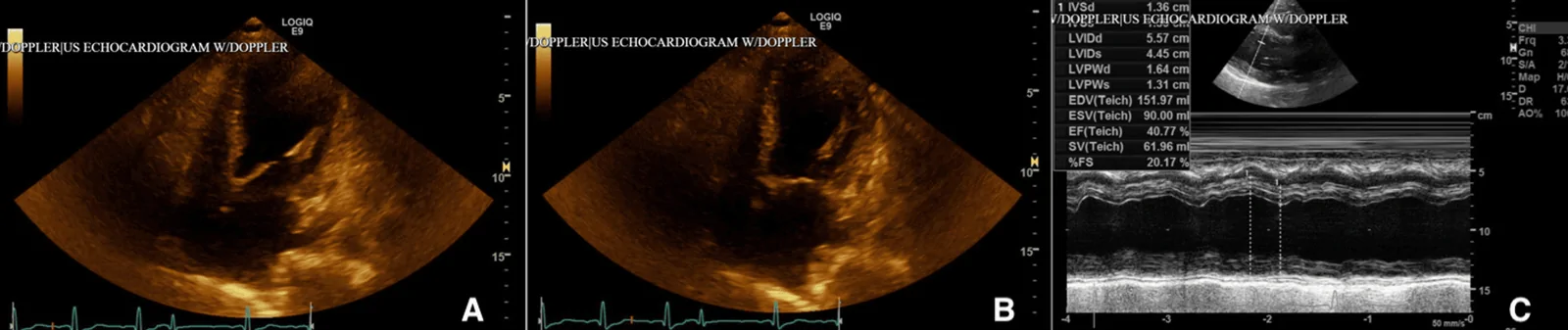
Follow-up transthoracic echocardiogram (A) End-diastolic apical four-chamber view. (B) End-systolic apical four-chamber view. (C) M-mode echocardiographic measurements demonstrating partial recovery of left ventricular systolic function, with a left ventricular internal diameter of 5.57 cm in diastole (LVIDd) and 4.45 cm in systole (LVIDs), and a calculated LVEF of 40.77%. LVIDd: left ventricular internal diameter in diastole, LVIDs: left ventricular internal diameter in systole, LVEF: left ventricular ejection fraction

Guideline-directed medical therapy for heart failure was optimized with carvedilol, spironolactone, and sacubitril/valsartan. The patient remained clinically stable and was discharged with outpatient cardiology follow-up. At six-month follow-up, transthoracic echocardiography demonstrated mild improvement in left ventricular systolic function, with the LVEF increasing to 40.77% (Figure 3).

## Discussion

In recent decades, recognition of antipsychotic-associated cardiotoxicity has increased substantially. Cardiovascular adverse effects associated with antipsychotic medications range from heart rate alterations and QT prolongation to myocarditis, heart failure, dilated cardiomyopathy, and sudden cardiac death. However, the underlying pathophysiology remains incompletely understood and is likely multifactorial [3,4].

The true incidence of olanzapine-associated cardiac injury remains unknown. Current evidence is largely limited to isolated case reports, pharmacovigilance signals, and small observational studies, suggesting that this complication is rare and potentially underrecognized [5].

Several mechanisms have been proposed to explain olanzapine-associated cardiotoxicity. Histopathologic studies have demonstrated findings including myocytolysis, myocardial necrosis, and inflammatory infiltrates, supporting a role for direct myocardial injury. Additional proposed mechanisms include inflammatory activation, oxidative stress, mitochondrial dysfunction, altered cellular energy metabolism, catecholamine-mediated toxicity, and cardiomyocyte apoptosis, all of which may contribute to ventricular remodeling and progressive systolic dysfunction. Studies have demonstrated effects on cardiac ion channel function, including potassium channel blockade, which may increase susceptibility to QT prolongation and ventricular arrhythmias. Furthermore, olanzapine is strongly associated with metabolic abnormalities such as weight gain, insulin resistance, dyslipidemia, and metabolic syndrome, which may further contribute to adverse cardiovascular outcomes [3,4,6-9].

Recent pharmacovigilance studies have reported cardiac toxicity during both short-term and long-term olanzapine exposure, suggesting that risk is not limited to the early phases of treatment.

A review of the literature identified a limited number of reports describing olanzapine-associated myocarditis or cardiomyopathy, underscoring both the rarity of this complication and the limited evidence currently available. The reports are summarized in Table 2.

**Table 2:** Clinical characteristics and outcomes of published cases of olanzapine-associated cardiac toxicity Cardiac presentation reflects the primary reported cardiac phenotype in each case. Causality between olanzapine exposure and cardiac injury was not definitively established in any report. LVEF: left ventricular ejection fraction, LV: left ventricle, GDMT: guideline-directed medical therapy

Author	Year	Patient demographics	Psychiatric disorder	Olanzapine dose and duration	Cardiac presentation/ diagnosis	Key findings (imaging/histopathology)	Outcome/reversibility
Puttegowda et al. [10]	2016	28/M	Bipolar disorder	5 mg/day for 10 years	Dilated cardiomyopathy	Four-chamber dilation, global biventricular dysfunction, LVEF 20%, normal coronary arteries	Partial recovery of cardiac function after olanzapine discontinuation and GDMT
Vang et al. [11] – case 1	2016	39/M	Schizophrenia	40 mg/day for 8 weeks	Eosinophilic myocarditis	Myocardial necrosis	Sudden cardiac death
Vang et al. [11] – case 2	2016	36/M	Schizophrenia	20-25 mg/day for 4 years	Eosinophilic myocarditis	Myocardial necrosis	Sudden cardiac death
Ioannou and Perera [12]	2022	65/F	Schizophrenia	10 mg/day for 2 years	Myocarditis	Biventricular enlargement	Normalization of ventricular function after drug withdrawal
Ito et al. [13]	2023	29/M	Schizoaffective disorder	10 mg/day for 2 weeks (following switch from clozapine)	Dilated cardiomyopathy	Severe heart failure exacerbation (EF 23%)	Substantial improvement in LVEF after treatment modification
Present case	2026	81/F	Bipolar disorder	5 mg/day for over 10 years	Dilated cardiomyopathy	Four-chamber dilation, global LV hypokinesis, LVEF 30-35%, normal coronary arteries	Partial recovery (LVEF improved to 40.77%) after olanzapine discontinuation and GDMT

Puttegowda et al. (2016) described a patient who developed nonischemic dilated cardiomyopathy after approximately 10 years of olanzapine treatment and was found to have normal coronary arteries, four-chamber dilation, and severe biventricular systolic dysfunction. Following discontinuation of olanzapine and initiation of guideline-directed heart failure therapy, partial recovery of cardiac function was observed. Our patient shared several similarities with this report, including bipolar disorder, low-dose olanzapine exposure for more than a decade, normal coronary arteries, and the development of severe nonischemic dilated cardiomyopathy [10].

Similarly, Vang et al. (2016) reported two fatal cases of olanzapine-associated myocardial injury. Both patients demonstrated myocardial necrosis and inflammatory myocardial involvement, ultimately resulting in sudden cardiac death. These cases suggest that olanzapine-associated cardiotoxicity may, in some patients, manifest as severe inflammatory myocardial injury with potentially fatal consequences [11].

More recently, Ioannou and Perera (2022) described a patient who developed severe biventricular dysfunction after approximately two years of olanzapine therapy. Cardiac magnetic resonance imaging confirmed myocarditis, and ventricular function normalized following discontinuation of olanzapine. This case provides further evidence that the cardiac injury may be reversible when recognized early and the offending agent is withdrawn [12].

Additional evidence for a potential association between olanzapine and cardiac side effects comes from the case reported by Ito et al. (2023). Worsening heart failure occurred after switching from clozapine to olanzapine in a patient with pre-existing dilated cardiomyopathy. Although multiple confounding factors limited causal attribution, the substantial improvement in LVEF following treatment modification further supports the possibility that olanzapine may contribute to myocardial dysfunction in susceptible individuals [13].

Tolu-Akinnawo et al. (2024) emphasized the diagnostic challenges associated with olanzapine-related cardiomyopathy and proposed that cardiac dysfunction may result from both direct myocardial toxicity and indirect metabolic effects. The authors highlighted the well-established association between olanzapine and metabolic abnormalities, including weight gain, insulin resistance, dyslipidemia, and metabolic syndrome, all of which may contribute to cardiac remodeling and heart failure [14].

We present the case of a patient who had received olanzapine 5 mg daily for more than 10 years. She exhibited newly diagnosed nonischemic dilated cardiomyopathy characterized by four-chamber dilation, global left ventricular hypokinesis, and an LVEF of 30-35%. Coronary angiography demonstrated normal coronary arteries. Consistent with previously published reports, our patient demonstrated partial recovery of ventricular function following discontinuation of olanzapine and initiation of guideline-directed medical therapy, with improvement in LVEF to 40.77% on follow-up echocardiography. The absence of obstructive coronary artery disease, the prolonged duration of olanzapine exposure, and partial recovery observed after olanzapine discontinuation and initiation of guideline-directed medical therapy raise suspicion for a potential association between olanzapine and the development of nonischemic dilated cardiomyopathy. This presentation was classified as a “possible” adverse drug reaction according to the Naranjo Adverse Drug Reaction Probability Scale (score of 4) [15].

Several alternative etiologies were considered during the diagnostic evaluation. Ischemic cardiomyopathy was unlikely given the absence of obstructive coronary artery disease on coronary angiography. Other potential causes of nonischemic cardiomyopathy, including valvular disease, thyroid dysfunction, and infectious etiologies, were considered based on the available clinical, laboratory, and imaging findings. Thyroid dysfunction was not supported by the normal thyroid-stimulating hormone level. Significant valvular disease was not identified on echocardiographic assessment, and there was no reported recent viral illness or other clinical evidence suggesting an acute infectious etiology.

Stress-induced cardiomyopathy (Takotsubo syndrome) was an important differential diagnosis. Takotsubo syndrome is characterized by transient left ventricular systolic dysfunction precipitated by physical or emotional stress that may clinically mimic an acute myocardial infarction. It occurs predominantly in postmenopausal women and is frequently triggered by significant life events. In the present case, the recent loss of her spouse represented a substantial emotional stressor [16].

Several findings were compatible with stress-induced cardiomyopathy. However, the classic echocardiographic pattern of Takotsubo syndrome consists of regional wall-motion abnormalities, typically involving apical and midventricular hypokinesis or akinesis with apical ballooning. This characteristic pattern was not observed in our patient. Instead, the present case demonstrated global left ventricular hypokinesis, four-chamber dilation, and persistent systolic impairment, findings that are less characteristic of classic Takotsubo syndrome. In addition, the coexistence of prolonged olanzapine exposure and similarities to previously published reports support consideration of a medication-related contribution to the development of cardiomyopathy [16].

This case highlights the diagnostic complexity of acute decompensated heart failure in older adults, in whom emotional stressors, chronic medication exposure, multimorbidity, and polypharmacy frequently coexist.

Current heart failure guidelines and the American Geriatrics Society Beers Criteria emphasize the importance of regularly reviewing potentially inappropriate medications and balancing therapeutic benefits against potential risks in geriatric patients. While olanzapine may be necessary for selected psychiatric conditions, clinicians should remain aware of uncommon but potentially serious cardiovascular complications. Cardiac biomarkers, electrocardiography, and echocardiography may be useful when clinically indicated, as early recognition of myocardial injury could facilitate timely intervention and potentially prevent progression to advanced cardiac dysfunction. A multidisciplinary approach involving primary care physicians, psychiatrists, geriatricians, and cardiologists may improve the identification of adverse drug effects and optimize patient outcomes [17].

Limitations

Although an extensive diagnostic evaluation was performed, including coronary angiography and serial cardiac assessment, cardiac magnetic resonance imaging and endomyocardial biopsy were not obtained. Consequently, definitive characterization of the underlying myocardial pathology was not possible. Thus, a causal relationship between olanzapine exposure and cardiomyopathy cannot be conclusively established. Importantly, these investigations are not routinely performed in all patients with newly diagnosed nonischemic cardiomyopathy, particularly once clinical stability has been achieved and the results are not expected to alter management. The Naranjo score of 4 supports classification as a possible adverse drug reaction, while also recognizing the potential contribution of concurrent clinical factors. While alternative etiologies, including stress-induced cardiomyopathy, cannot be completely excluded, the combination of prolonged olanzapine exposure, absence of obstructive coronary artery disease, partial recovery following drug withdrawal, and consistency with previously published reports highlights a possible adverse drug effect that warrants further investigation and awareness among clinicians.

## Conclusions

Olanzapine-associated cardiomyopathy is a rare and likely underrecognized clinical entity. This case highlights the diagnostic challenges of heart failure in older adults, where multimorbidity, polypharmacy, and competing risk factors may obscure medication-related adverse effects. Clinicians should maintain a broad differential diagnosis and consider potential olanzapine-associated cardiac toxicity in patients who develop dyspnea or other signs and symptoms of heart failure during treatment. Early recognition, prompt discontinuation of the suspected causative agent, and initiation of guideline-directed heart failure therapy may help prevent progression to severe myocardial damage. Cardiac biomarkers, electrocardiography, and echocardiography may facilitate earlier detection of myocardial injury, and periodic cardiac assessment may be warranted in selected patients receiving long-term antipsychotic therapy, particularly those with preexisting cardiovascular disease.

This report raises awareness of a potentially underrecognized cardiovascular complication of prolonged olanzapine use. More broadly, it underscores the importance of comprehensive medication reviews, shared decision-making, and close interdisciplinary collaboration when managing older adults exposed to complex medication regimens.
